# AN EXPLORATION OF A DIGITAL FALL PREVENTION PROGRAM: “KEEP ON KEEP UP” FOR OLDER ADULTS IN A CHINESE CULTURAL CONTEXT

**DOI:** 10.1093/geroni/igae098.1078

**Published:** 2024-12-31

**Authors:** Yimin Tang, Sarah Smith, Sorrel Burden, Emma Stanmore

**Affiliations:** University of Manchester, Manchester, England, United Kingdom; University of Manchester, Manchester, England, United Kingdom; University of Manchester, Manchester, England, United Kingdom; University of Manchester, Manchester, England, United Kingdom

## Abstract

The use of digital health interventions can be effective in enabling and maintaining healthy behaviours to help prevent falls and improve physical function in older adults. However, scarce research has been conducted with specific cultural and minority groups living in a cross-cultural setting. The aim of this study was to explore Chinese older adults’ and stakeholders’ experience, needs and preferences related to using digital health interventions, in particular the Keep On Keep Up-Chinese (KOKU-C) falls prevention program (translated to Mandarin language). This research used think-aloud as a novel approach in interviews and was completed within a cross-cultural context to better improve user-experience, engagement and digital inclusion of Chinese older adults living in the UK. A sample of Chinese older adults 55 to 95 years of age (N=40) living in Manchester and stakeholder representatives including healthcare professionals, caregivers, community partners and researchers (N=12) have been interviewed. Results from thematic analysis found that while there are opportunities to better enhance the digital health interventions tailored to the Chinese cultural context, the KOKU-C program can still provide valuable information as well as balance and strength training to help support fall-related health outcomes and improve their quality of life. These important findings point to the need for more research taking cultural considerations into account when co-designing digital health applications. The potential benefits of this study provide a possible solution for better acceptability, engagement and interaction in digital health interventions with Chinese older populations.

